# Comparing traditional natural language processing and large language models for mental health status classification: a multi-model evaluation

**DOI:** 10.1038/s41598-025-08031-0

**Published:** 2025-07-06

**Authors:** Thomas Kallstenius, Andrea Johansson Capusan, Gerhard Andersson, Adam Williamson

**Affiliations:** 1 Department of Biomedical and Clinical Sciences, Center for Social and Affective Neuroscience, Linköping University, Linköping, Sweden; 2 Trädtopp, Tervuren, Belgium; 3 Department of Behavioural Sciences and Learning, Department of Biomedical and Clinical Sciences, Linköping University, Linköping, Sweden; 4 Department of Clinical Neuroscience, Psychiatry Section, Karolinska Institutet, Stockholm, Sweden; 5 International Clinical Research Center, St. Anne’s University Hospital Brno, 60200 Brno, Czech Republic

**Keywords:** Mental health classification, Natural language processing, Large language models, Text classification, Machine learning in psychiatry, Computational psychiatry, Computational neuroscience, Emotion

## Abstract

The substantial increase in mental health disorders globally necessitates scalable, accurate tools for detecting and classifying these conditions in digital environments. This study addresses the critical challenge of automated mental health classification by comparing three distinct computational approaches: (1) Traditional Natural Language Processing (NLP) with advanced feature engineering, (2) Prompt-engineered large language models (LLMs), and (3) Fine-tuned LLMs. The dataset consisted of over 51,000 publicly available text statements from social media platforms, tagged with seven mental health conditions: Normal, Depression, Suicidal, Anxiety, Stress, Bipolar Disorder, and Personality Disorder. The dataset was stratified into training, validation, and test sets for model evaluation. The primary outcome was classification accuracy across these seven mental health conditions. Additional metrics like precision, recall, and F1-score were analyzed. We compared the results of the three computational approaches and overfitting was monitored through validation loss across epochs for the fine-tuned LLM. The NLP model with advanced feature engineering achieved an overall accuracy of 95%, surpassing both the prompt-engineered LLM (65%) and the fine-tuned LLM (91%). This model performed exceptionally well in terms of accuracy and precision. While fine-tuning for three epochs yielded optimal results, further training led to overfitting and decreased performance. This study demonstrates the significant benefits of applying advanced text preprocessing and feature engineering techniques to traditional NLP models, alongside fine-tuning LLMs, such as GPT-4o-mini, for mental health classification tasks. The results clearly indicate that off-the-shelf LLM chatbots using prompt engineering are inadequate for mental health classification, performing 30% points below specialized NLP approaches. Despite the popularity of general-purpose LLMs, specialized approaches remain superior for critical healthcare applications like mental health classification.

## Introduction

Mental health disorders not only cause considerable suffering and economic costs to patients, families, and society but also constitute a significant proportion of the global burden of disease^1^. The actual prevalence of mental health conditions is, however, often underestimated, making the accurate detection and classification of these conditions critical for timely intervention and improved outcomes^2^. With the rise of digital health platforms and the increasing amount of mental health-related content shared online, there is an opportunity to leverage machine learning to support mental health professionals in identifying and categorizing conditions based on language use^3^. Advanced natural-language-processing (NLP) models—particularly large language models (LLMs)—have shown considerable promise in performing complex tasks such as text classification, sentiment analysis^4^.

The initial hypothesis of this study is that specialized, task-optimized approaches—traditional NLP with domain-specific feature engineering or LLMs fine-tuned on in-domain data—will outperform generic “zero-shot” LLM prompting for mental-health classification. Whereas general-purpose LLMs possess broad linguistic knowledge, domain-specific preprocessing and fine-tuning should yield higher precision and recall for nuanced clinical categories. This perspective contrasts with the recent trend of substituting specialized systems with off-the-shelf LLMs for many NLP tasks.

In a previous study, neuroimaging, EEG or genetic data were analyzed to predict mental health outcomes, offering a passive and continuous monitoring form^5^. However, this work did not address the direct analysis of textual data, such as social media posts or conversations with chatbots, which have become an increasingly rich source for understanding mental health trends and conditions^6^. Several other publications have explored the potential of utilizing deep learning models and NLP to identify psychiatric disorders based on user content on social media^3,7^. Many of these studies have focused on deep neural networks (DNNs) and convolutional neural networks (CNNs), demonstrating their potential to detect psychiatric conditions and suggesting that, in the future, such models could assist in alerting users before clinic visits, thereby improving access to mental health services and diagnostic accuracy.

Early clinical-text mining relied on rule-based pipelines such as MetaMap and cTAKES, which map free-form notes to the Unified Medical Language System (UMLS) ontology^8,9^. More recently, Dalal et al.^10^ showed that integrating PHQ-9 clinical-practice guidelines with a cross-attention transformer yields explainable depression classification. Another paper introduced a “knowledge-infusion” strategy that embeds disorder ontologies directly into the model for transparent predictions^11^. Today, transformer language models pre-trained on biomedical data—BioBERT, ClinicalBERT and the psychiatry-focused MentalBERT—achieve state-of-the-art performance on tasks such as suicide-risk note classification^12,13^.

Despite these advances, direct comparisons between traditional feature-rich NLP models and modern LLMs on the same mental-health dataset remain scarce. The present study uses traditional NLP models combined with advanced text preprocessing techniques, benchmarking their performance against modern LLMs in their pre-trained state and after fine-tuning. Fine-tuning involves adapting a pre-trained model to a specific task or dataset by continuing its training on task-specific data, enabling the model to retain its broad knowledge from pre-training while acquiring domain-specific insights for the new application^14^. This comparison allows us to evaluate how well traditional machine learning techniques perform in relation to state-of-the-art LLMs, providing valuable insights into the effectiveness and simplicity of various mental health pattern detection approaches in user-generated content. Using a large dataset of over 52,000 social media posts labelled with seven mental health conditions, this study seeks to identify the most effective and scalable method for mental health classification. The findings aim to inform the integration of AI tools into mental health care, supporting early detection, improved diagnostic accuracy, and broader access to services.

## Methods

### Dataset

The dataset used in this study comprises 52,681 unique text statements, each labelled with one of seven mental health statuses: Normal, Depression, Suicidal, Anxiety, Stress, Bipolar Disorder, and Personality Disorder. This dataset was curated from publicly available mental health-related datasets on Kaggle which draw data from social media platforms such as Reddit and Twitter, where mental health discussions are prevalent. It integrates contributions from a variety of sources, including datasets such as Depression Reddit Cleaned, which focuses on depression-related discussions on Reddit; Suicidal Tweet Detection, aimed at identifying potential Suicidal ideation on Twitter; and Human Stress Prediction, which detects Stress indicators in text.

Dataset: https://www.kaggle.com/datasets/suchintikasarkar/sentiment-analysis-for-mental-health/data.

Although the Kaggle dataset does not explicitly specify the data collection period, it is likely drawn 2018 to 2022, when similar datasets became available on Kaggle and other platforms. The data were gathered over different timeframes and aggregated from various sources, providing a broad and diverse representation of mental health-related conversations on social media.

The dataset consists of English-language posts. The open nature of the platforms used suggests contributions from users worldwide, particularly those where mental health discussions in English are common.

The labels assigned to each text entry reflect either the user’s explicitly stated or inferred mental health condition at the time of posting. Notably, these labels do not correspond to clinical diagnoses made by healthcare professionals but instead represent self-reported symptoms or discussions about mental health. As a result, the dataset should not be interpreted as representing medically confirmed conditions. Users engaging in mental health discussions on platforms like Reddit and Twitter are often part of self-help groups, supportive communities, or general forums where mental health is a primary topic. Therefore, the text may describe personal experiences, symptoms, or inquiries without clinical validation.

The dataset may include multiple statements from the same participants, particularly for posts sourced from Reddit. Users on Reddit tend to contribute repeatedly within specific subreddits, such as those focused on Depression or Anxiety. However, the dataset does not explicitly track whether posts can be linked to individual users over time. While this may mean that some posts reflect an individual’s mental health across multiple instances, the anonymity of these platforms means there is no definitive link between posts and specific users.

### Preprocessing

To ensure consistency and accurate text representation, the following preprocessing steps were applied:

1. Text normalization: Text was converted to lowercase, punctuation was removed, URLs and numbers were filtered out, and text was tokenized to standardize the input data.
2. Stopword removal: Common English stopwords were removed using the NLTK library to focus on the most relevant terms for classification.
3. Text vectorization: A Term Frequency-Inverse Document Frequency (TF-IDF) Vectorizer with bigram features was used to convert the text into numerical form. The vectorizer was set to a maximum of 10,000 features, with an n-gram range of 1 to 2, allowing for the capture of word sequences and co-occurrence patterns.
4. Data augmentation: TextBlob was used to perform back-translation to enhance the dataset, where the text was translated into French and then back into English. This step introduced additional linguistic variations of the original statements, particularly useful for improving the model’s robustness, especially in underrepresented categories like personality disorder.

### Stratified train-test split

**Fig. 1:**
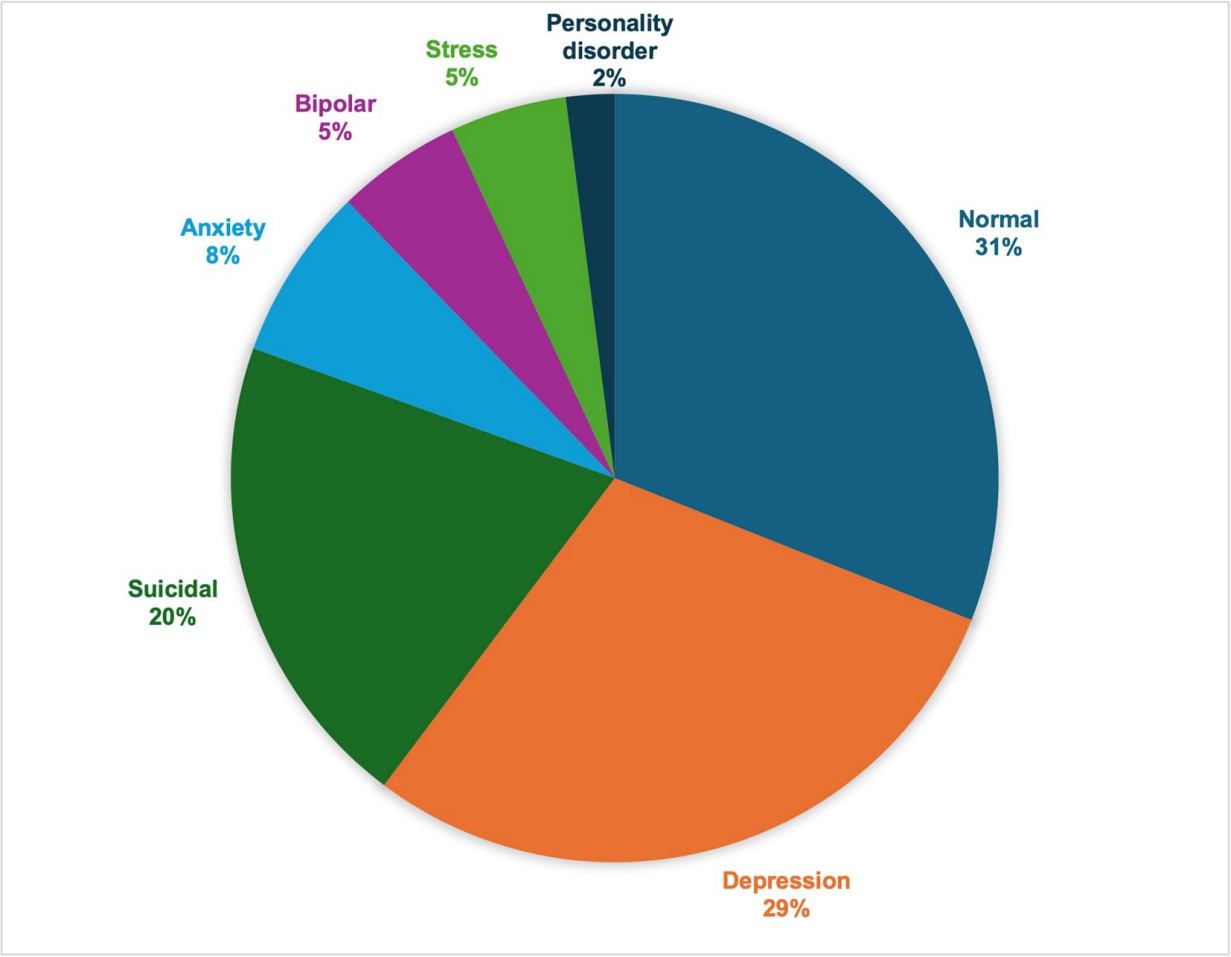
Distribution of mental health status labels in the dataset.

One of the primary challenges posed by this dataset is the issue of class imbalance, where conditions such as Depression and Suicide are overrepresented. At the same time, less common labels like Personality Disorder are underrepresented, as shown in Fig. 1. A stratified train-test split was applied to the dataset to ensure a balanced representation across all classes and avoid data leakage. The dataset was split into:

- Training Set: 80% of the dataset for all models.
- Validation Set: Used only for the fine-tuned LLM (10% of the dataset).
- Test Set: 20% of the dataset for evaluating the traditional NLP model and prompt-engineered LLM, and 10% of the dataset for evaluating the fine-tuned LLM.

The validation set was necessary for the fine-tuned LLM because fine-tuning involves optimizing a pre-trained model on task-specific data. This requires iterative updates to the model’s parameters, and the validation set helps monitor performance during training to avoid overfitting. The validation set enables the selection of the best-performing version of the fine-tuned LLM by assessing its generalization to unseen data, ensuring that the model retains its ability to generalize beyond the training set.

In contrast, the traditional NLP models and prompt-engineered LLM were not fine-tuned and, therefore, did not require a separate validation set. For these models, evaluations were performed directly using the test set. While the test set proportions differ between the models (20% for NLP and prompt-engineered LLM vs. 10% for fine-tuned LLM), the stratified nature of the sampling ensures that all test sets maintained representative distributions of the seven mental health conditions, allowing for valid performance comparisons.

By using this stratified approach across all dataset splits, the study minimized the risks of bias that can arise due to class imbalance, ensuring that the models were trained on a representative distribution of data and providing a robust basis for performance evaluation.

This chart illustrates the distribution of the 52,681 text statements across the seven mental health statuses used in this study: Normal, Depression, Suicidal, Anxiety, Stress, Bipolar Disorder, and Personality Disorder. The distribution highlights the imbalance across categories, with some conditions (e.g., Depression and Suicidal) being more frequently represented than others (e.g., Personality Disorder, Stress and Bipolar Disorder). This imbalance presents a potential challenge for classification models, particularly in underrepresented classes.

### Model selection

Three different models were evaluated:

1. Prompt-engineered LLM: We used a pre-trained GPT-4o-mini-2024-07-18 model. The model was applied using prompt engineering techniques, where specific prompts were designed to extract relevant mental health classifications without additional model training. No further task-specific tuning was applied.
2. Fine-tuned LLM: A fine-tuned version of GPT-4o-mini-2024-07-18 was trained on the mental health dataset. Fine-tuning was conducted over 1 to 4 epochs, allowing the model to adapt to task-specific linguistic patterns found in mental health statements.
3. NLP model with advanced preprocessing: We implemented an NLP approach using advanced preprocessing techniques, including text normalization and stopword removal. This NLP model uses TF-IDF vectorization and an SVM with a Radial Basis Function (RBF) kernel, including a regularization parameter C. It was tuned to maximize accuracy, using the TF-IDF vectorization with up to 10,000 features.

### Evaluation metrics

The models were evaluated based on classification accuracy. The accuracy was calculated by comparing predicted labels with actual labels for each test set statement. Additional metrics such as precision, recall, and F1-score were recorded for a more comprehensive performance evaluation, defined as follows:

- Accuracy: refers to the overall effectiveness of a model in correctly predicting mental health statuses. It is the ratio of correct predictions to the total number of predictions. In other words, it measures how often the model is right across all categories.
  1 $${\text{Accuracy = (TP + TN)/(TP + TN + FP + FN) }}$$
  where
  - TP = True Positives (correctly predicted positive instances).
  - TN = True Negatives (correctly predicted negative instances).
  - FP = False Positives (incorrectly predicted as positive).
  - FN = False Negatives (incorrectly predicted as negative).
- Precision: Precision focuses on the quality of the positive predictions. It answers the question: “When the model predicts a certain condition, how often is it correct?” A high precision means that it is usually correct when the model predicts a specific mental health condition.
  2 $${\text{Precision = TP/(TP + FP) }}$$
- Recall: Recall measures how well the model identifies actual cases of a mental health condition. It answers the question: “Out of all the actual cases of a condition, how many did the model correctly identify?” A high recall indicates that the model is good at detecting true cases, even if there are some false positives.
  3 $${\text{Recall = TP/(TP + FN) }}$$
- F1-score: The F1-score is the balance between precision and Recall, providing a single number that accounts for both. It is beneficial when there is an uneven class distribution or when the cost of false positives and false negatives differs. The F1-score is high when both precision and Recall are high, meaning the model is accurate in its predictions and good at identifying real cases.
  4 $${\text{F1 - Score = 2 }} \times {\text{ (Precision }} \times {\text{ Recall)/(Precision + Recall) }}$$

### Comparison with clinical evaluation metrics

In clinical diagnostics, similar metrics are used, but they are referred to with different names and sometimes have slight variations in interpretation^15^:

- Precision in machine learning corresponds to Positive Predictive Value (PPV) in clinical terms. Both metrics measure the proportion of positive predictions that are actually correct. In other words, precision/PPV evaluates the likelihood that a positive result truly reflects the presence of a condition (e.g., if the model predicts Depression, how often is the person actually depressed?).
- Recall in machine learning is equivalent to sensitivity in clinical settings. Both metrics assess how well the model/test identifies actual cases of a condition. High Recall or Sensitivity means that the model is good at identifying people with the condition, minimizing the chance of false negatives (missing real cases).
- The F1 score, which balances precision and Recall, has no exact clinical equivalent but can be considered a balance between Sensitivity and PPV. This makes it particularly useful in cases of class imbalance, similar to how clinicians might balance the importance of catching all true cases (high Sensitivity) with avoiding over-diagnosis (high PPV).
- Other clinical terms, such as Specificity, Negative Predictive Value (NPV) and Likelihood Ratios, are typically not accounted for in standard machine learning metrics.

## Results

This section presents the performance of the three models evaluated. The models were assessed using accuracy, precision, recall, and F1-score as the primary evaluation metrics. The results are detailed below, particularly emphasizing how the stratified train-test split affected model performance across different mental health conditions.

**Fig. 2:**
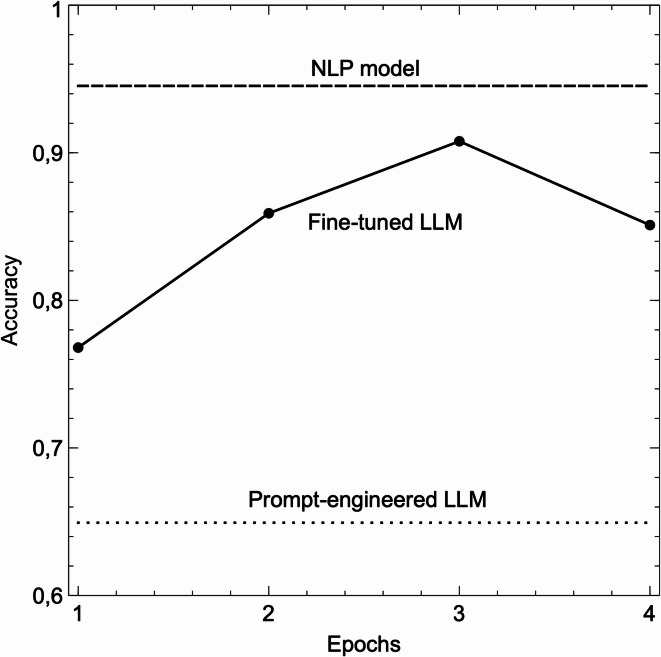
Accuracy comparison of three models for mental health classification:

This figure compares the accuracy of three different computational approaches used in the study: (1) Prompt Engineering with GPT-4o-mini, (2) NLP Model with advanced preprocessing, and (3) Fine-tuned GPT-4o-mini. An epoch represents one complete cycle through the entire training dataset, where the model adjusts its internal parameters to better predict the correct labels.

### Prompt-engineered LLM model results

The base GPT-4o-mini model, using prompt engineering without further task-specific tuning, achieved an overall accuracy of 65%, see Fig. 2. Table 1 presents the full classification report, and Table 2 shows the confusion matrix. Its performance was notably lower across several categories:

- Precision ranged from 25% (stress) to 88% (normal).
- Recall was highest for suicidal tendencies (80%) but was low for conditions like stress (48%).
- The model struggled to identify underrepresented conditions, such as personality disorder, with a precision of only 28% and a recall of 34%.

This result indicates that while leveraging pre-trained knowledge bases, prompt engineering without fine-tuning may be insufficient for the specific nuances of mental health classification.

**Table 1:** Classification report for base GPT-4o-mini model.

Class	Precision	Recall	F1-score
Depression	0.72	0.49	0.58
Normal	0.88	0.73	0.80
Suicidal	0.60	0.80	0.69
Bipolar	0.83	0.57	0.68
Stress	0.25	0.48	0.33
Anxiety	0.49	0.77	0.60
Personality disorder	0.28	0.34	0.31

Overall accuracy: 65%.

**Table 2:** Confusion matrix for base GPT-4o-mini model (in %), the y-axis represents the actual (true) labels, while the x-axis represents the predicted labels produced by the model:

	Depression	Normal	Suicidal	Bipolar	Stress	Anxiety	Personality disorder
Depression	49.06%	3.35%	31.67%	1.23%	4.51%	8.55%	1.64%
Normal	4.83%	73.41%	2.19%	0.16%	12.77%	3.48%	2.28%
Suicidal	12.05%	2.52%	79.92%	0.35%	2.47%	1.78%	0.89%
Bipolar	11.30%	8.29%	5.65%	56.88%	9.23%	6.21%	2.44%
Stress	9.33%	7.51%	4.06%	1.01%	48.28%	24.34%	5.57%
Anxiety	2.08%	8.86%	1.52%	0.97%	9.14%	76.94%	0.28%
Personality disorder	22.61%	10.05%	8.04%	0.00%	7.04%	17.59%	34.17%

### Fine-tuned LLM model results

Fine-tuning the GPT-4o-mini model improved performance significantly, achieving an accuracy of 91% after three epochs, as seen in Fig. 2. The detailed classification report is shown in Table 3, and Table 4 shows the confusion matrix.

- Precision remained high for common categories such as Normal (99%) and Anxiety (97%) but was lower for more complex categories like Suicidal tendencies (83%) and Depression (85%).
- Recall was strongest for Personality Disorder (94%) and Bipolar Disorder (93%), showing the model’s capability to detect true cases in underrepresented categories due to the balanced data provided by the stratified split.

However, when the model was trained for a fourth epoch, accuracy dropped to 85%, indicating overfitting. This highlights the importance of careful hyperparameter tuning when fine-tuning large language models for specific tasks.

**Table 3:** Classification report for fine-tuned GPT-4o-mini model.

Class	Precision	Recall	F1-score
Depression	0.85	0.88	0.87
Normal	0.99	0.99	0.99
Suicidal	0.83	0.80	0.82
Bipolar	0.98	0.93	0.95
Stress	0.90	0.92	0.91
Anxiety	0.97	0.93	0.95
Personality disorder	0.92	0.94	0.93

Overall accuracy: 91%.

**Table 4:** Confusion matrix for fine-tuned GPT-4o-mini model (in %).

	Depression	Normal	Suicidal	Bipolar	Stress	Anxiety	Personality disorder
Depression	88.33%	0.31%	10.81%	0.10%	0.10%	0.14%	0.10%
Normal	0.19%	98.55%	0.19%	0.00%	0.84%	0.19%	0.00%
Suicidal	19.76%	0.25%	79.85%	0.00%	0.05%	0.00%	0.00%
Bipolar	4.33%	0.19%	0.19%	93.03%	0.75%	0.56%	0.94%
Stress	1.42%	4.26%	0.20%	0.00%	92.10%	1.42%	0.61%
Anxiety	1.80%	1.25%	0.28%	1.11%	1.94%	92.63%	0.83%
Personality disorder	3.02%	0.50%	0.50%	0.50%	0.00%	1.01%	94.47%

### NLP model results

The NLP model, using TF-IDF vectorization and an SVM with an RBF kernel, achieved the highest accuracy of 95% across all conditions. Table 5 presents the full classification report, and Table 6 shows the confusion matrix.

**Fig. 3:**
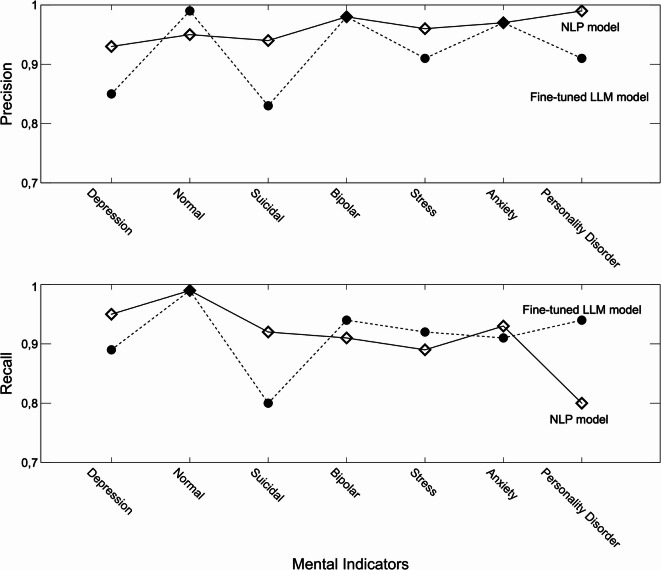
Precision for the NLP and fine-tuned model and Recall for the NLP and fine-tuned model.

- Precision was highest for conditions like Personality Disorder (99%) and Bipolar Disorder (98%), demonstrating strong performance even in categories with fewer data points, see Fig. 3.
- Recall was also strong, particularly for Depression (95%) and Anxiety (93%), indicating the model’s ability to identify true positives reliably, while the result for Personality Disorder (80%) was significantly lower, see Fig. 3.

The consistent performance of the NLP model, particularly in conditions that are commonly overrepresented (e.g., Depression and Anxiety), shows the strength of traditional approaches when combined with advanced feature engineering.

**Table 5:** Classification report for NLP modelclassification report for advanced NLP.

Class	Precision	Recall	F1-score
Anxiety	0.97	0.93	0.95
Bipolar	0.98	0.91	0.94
Depression	0.93	0.95	0.94
Normal	0.95	0.99	0.97
Personality disorder	0.99	0.80	0.89
Stress	0.96	0.89	0.92
Suicidal	0.94	0.92	0.93

**Table 6:** Confusion matrix for the NLP model.

	Depression	Normal	Suicidal	Bipolar	Stress	Anxiety	Personality disorder
Depression	91.76%	0.05%	0.00%	0.05%	1.64%	6.41%	0.09%
Normal	0.37%	88.76%	0.00%	0.94%	6.55%	3.37%	0.00%
Suicidal	0.00%	0.42%	80.42%	0.00%	12.92%	6.25%	0.00%
Bipolar	0.00%	0.39%	0.00%	93.44%	3.47%	2.57%	0.13%
Stress	0.18%	0.21%	0.00%	0.08%	98.85%	0.64%	0.03%
Anxiety	3.31%	0.16%	0.06%	0.29%	0.94%	94.97%	0.26%
Personality disorder	0.70%	0.00%	0.00%	0.35%	4.53%	3.57%	90.86%

We integrated the NLP model into a web-based application built around a recommendation and advice engine. This application interacts with users by posing a minimum of five open-ended questions, generating an aggregated result based on the user’s responses. The NLP model provides classification outcomes with confidence scores across various mental health indicators. offering insights based on the cumulative input.

**Fig. 4:**
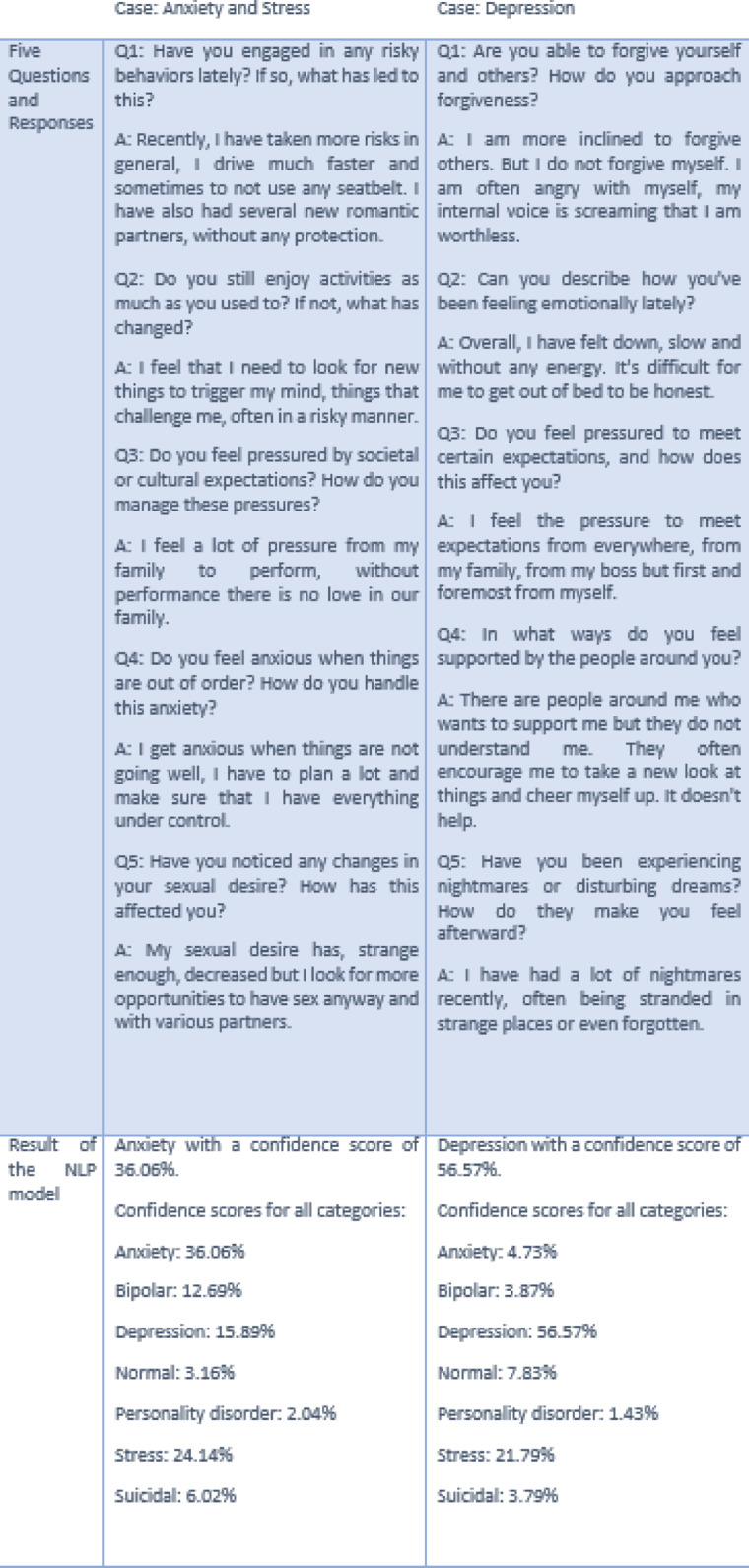
Qualitative examples from the AI-based web application. This figure presents two different classification outcomes generated by the NLP model within the AI-driven web application. These outcomes are based on conversations between clients and the application. The model evaluates the aggregated responses from a series of random open-ended questions, with a minimum of five questions per session. These examples highlight the system’s ability to analyze and classify user inputs based on natural language processing.

As illustrated in Fig. 4, the application showcases examples of how the system processes responses and categorizes mental health statuses. The examples in the figure use shorter text inputs for clarity, but in real-world use, the model processes longer, more complex responses. This approach enhances the accuracy and reliability of the classification results, making the application a valuable tool for preliminary mental health assessments in a safe and anonymous environment.

## Discussion

The results of this study highlight the advantages of using both NLP models and fine-tuning LLMs for specialized tasks, particularly in the classification of mental health conditions based on text data. In this study, the NLP model outperformed all other approaches, achieving the highest accuracy, followed by the fine-tuned GPT-4o-mini model and the prompt-engineered GPT-4o-mini model.

### Performance of the models

The prompt-engineered GPT-4o-mini model performed considerably worse than the NLP and fine-tuned versions, achieving only 65% accuracy most likely due to the lack of domain-specific adaptation: While pre-trained LLMs possess broad knowledge, they have not been specifically optimized for the nuanced language and contextual cues in mental health expressions.

Related to this is the challenge of contextual ambiguity. Mental health expressions often contain subtle linguistic patterns that can be ambiguous without proper context^16,17^. Without fine-tuning on domain-specific data, the model struggled to differentiate between closely related conditions like anxiety and depression with overlapping yet distinct language patterns^16^.

Fine-tuning allowed the GPT-4o-mini model to adapt to the specific linguistic patterns in the mental health dataset. The fine-tuned LLM model performed well, particularly in handling complex categories like Personality Disorder, which generalized models often misclassify.

The fine-tuned LLM model achieved peak accuracy at 91% after 3 epochs, but performance declined to 85% after 4 epochs, probably due to overfitting. Overfitting occurs when a model becomes too closely aligned with the training data, capturing noise or irrelevant details, which reduces its ability to generalize to unseen data^18^. The training curves in Fig. 1 illustrate this, showing that while training accuracy improved, validation accuracy decreased after 3 epochs. This emphasizes the importance of careful hyperparameter tuning and monitoring during fine-tuning to avoid overfitting.

The NLP model demonstrated superior accuracy, achieving the highest performance of 95% across the board, particularly excelling in common mental health conditions such as Anxiety and Depression. While it faced some challenges when classifying underrepresented conditions like Personality Disorder, its overall accuracy outshined the fine-tuned model. Additionally, the NLP model has several practical advantages, including local operation without reliance on external servers, which enhances privacy for sensitive applications. Moreover, its smaller size and efficiency make it ideal for resource-constrained environments and real-time applications, where speed and interpretability are crucial.

### Comparison of the NLP model and fine-tuned LLM model

The use of a stratified train-test split in this study ensures that the evaluation of the NLP and fine-tuned models is not biased by class imbalances. This approach proportionally represents all mental health conditions, including underrepresented ones such as bipolar and personality disorders, across the training, validation, and test sets. As a result, both models were trained and tested on balanced datasets, which led to more reliable and meaningful performance metrics.

As shown in Fig. 3, the NLP model shows consistently high precision across most conditions, particularly for Personality Disorder (99%), Bipolar Disorder (98%), and Suicidal tendencies (94%). The stratified split allows the NLP model to maintain strong performance even in conditions with fewer data points, such as Personality Disorder and Stress. This suggests that the NLP model excels in reducing false positives across various conditions, including rare ones.

The fine-tuned model, while showing high precision for more common categories like Normal (99%), exhibits lower precision in conditions like Suicidal tendencies (83%), Stress (91%) and Personality Disorder (91%). The stratified approach ensures that these differences are not due to class imbalances but rather reflect inherent differences in the models’ capacity to generalize across conditions.

Concerning recall, Fig. 3, the fine-tuned model demonstrates its strength in capturing true positives for underrepresented conditions, particularly for Personality Disorder (94%) and Bipolar Disorder (94%). The stratified split ensures that the fine-tuned model had adequate exposure to these categories during training, enabling it to detect a higher proportion of true cases compared to the NLP model, which achieved 80% recall for Personality Disorder.

For conditions like Depression and Suicidal tendencies, the NLP model performs better in terms of Recall, reaching 95% and 92%, respectively. This indicates that the NLP model is more adept at identifying true positives in these critical categories, despite the balanced training. While robust in Recall for other conditions, the fine-tuned model shows a lower recall for Suicidal tendencies (80%), which could highlight challenges in identifying this complex condition with high accuracy.

Apart from the superior accuracy and more consistent precision and recall, the NLP model offers several significant advantages over fine-tuned models, including:

1. Local data processing:

Unlike large LLMs that may require external servers or cloud computing, the NLP model processes data entirely on local devices. This enhances privacy and security, which are crucial in sensitive domains such as mental health.

2. Confidence scores:

The NLP model provides more interpretable confidence scores across all mental health indicators, offering a transparent view of the model’s certainty in its classifications. This is an important feature in real-world applications where users must trust the model’s predictions.

3. Efficiency:

The NLP model is smaller and more efficient than LLMs, making it capable of running on standard hardware. This makes it an attractive option for real-time applications in resource-constrained environments, where speed and computational efficiency are crucial.

4. Holistic assessment:

By providing a distribution of confidence scores across multiple mental health indicators, the NLP model aligns with the growing trend of treating individuals as multifaceted rather than assigning a single label. This approach emphasizes the interconnectedness of mental health traits, contributing to a more nuanced and person-centered understanding of mental health.

### Meaningful metrics in mental health classification

When evaluating models for mental health classification, certain metrics hold particular clinical significance and should be weighted more heavily depending on the specific condition and intended application.

Recall, i.e. sensitivity in clinical research, becomes the most critical metric for high-risk conditions such as suicidal ideation, as the cost of missing a true case (false negative) could be life-threatening. The NLP model’s higher Recall for suicidal tendencies (92% vs. 80% for the fine-tuned LLM) represents a significant advantage in this safety-critical category.

Precision/PPV becomes more important for resource-intensive interventions, to avoid unnecessary allocation of limited clinical resources. This is particularly relevant for specialized treatments that require significant time from mental health professionals.

Accuracy provides a clear overall assessment of model performance across all categories, making it particularly valuable for general screening tools. F1-score complements accuracy by providing a balanced assessment when the model is intended for initial screening, where both false positives and false negatives have meaningful consequences. The NLP model’s superior accuracy (95% vs. 91% for the fine-tuned LLM) and consistent F1-scores above 89% across all conditions make it particularly suitable for general screening applications.

For this specific mental health dataset with its inherent class imbalance, the F1-score emerges as particularly informative as it accounts for both recall (sensitivity) and precision (PPV) and challenges posed by conditions like Personality Disorder, comprising only a small portion of the dataset.

However, when implementing such systems in real-world clinical settings, threshold optimization should be condition-specific and account for the trade-off between sensitivity and specificity^19^.

### Human performance and model comparison

A recent study^20^ tested whether NLP techniques could identify cognitive distortions in text messages between clients with serious mental illness and therapists at a level comparable to trained clinicians, achieving F1-scores between 41% and 63% for the latter. While not directly comparable due to differing datasets and indicators, our NLP model achieved significantly higher F1-scores (89–97%, as seen in Table 5), suggesting it can match or exceed human performance in specific text-based classification tasks. However, AI lacks the nuanced judgment, contextual understanding, and empathy inherent to human expertise. Integrating AI with human oversight offers a powerful hybrid approach, where AI provides scalable, efficient assessments and flags potential risks, while clinicians incorporate broader contextual factors for empathetic, well-rounded care. Together, this synergy enhances early detection, intervention, and equitable access to mental health support.

### Neurodiverse considerations

It is important to highlight that the dataset used in this study does not distinguish between neurotypical and neurodiverse individuals. Mental health expressions can differ significantly among neurodiverse populations, such as those with autism or ADHD, compared to neurotypical individuals. These variations in communication and emotional expression are crucial when assessing mental health conditions, as current models may not accurately capture the unique ways in which neurodiverse individuals express their experiences.

Given this limitation, there is a pressing need to develop datasets and fine-tune models that are more inclusive and sensitive to neurodiverse expressions of mental health. Failing to account for these differences risks introducing biases, leading to potential misclassification or inadequate support for neurodiverse populations. Future work should focus on creating more representative datasets and adapting models to ensure that mental health classification systems are equitable and accurate for all individuals, regardless of neurotype. This will help to prevent biases and improve the reliability of mental health tools in diverse populations.

### Strengths and limitations

This study has several strengths. It uses a large and diverse dataset of over 52,000 social media posts, ensuring robust training and evaluation across various mental health conditions. The methodological transparency, including detailed preprocessing and evaluation steps, allows reproducibility and future advancements. Additionally, the NLP model’s simplicity, efficiency, and interpretability make it adaptable for real-world applications, including resource-constrained environments, and its ability to provide confidence scores enhances its practical utility for healthcare professionals.

However, the study has limitations. The dataset is based on self-reported or inferred mental health indicators from social media, which lack clinical validation and may introduce bias. It does not account for neurodiverse populations, potentially underrepresenting or misclassifying unique expressions of mental health. The reliance on text alone excludes other contextual or multimodal signals crucial for comprehensive mental health assessments. Furthermore, while prompt-engineered LLMs showed limited performance in this study, advancements in these models may address current shortcomings in the future.

### Future work

The findings and limitations of this study suggest several promising avenues for future research:

1. Comprehensive LLM benchmarking.

Future studies should benchmark a wider spectrum of language models, including (i) newer general-purpose LLMs (e.g., GPT-4o-mini, Gemini-1.5-Pro), (ii) domain-specialised medical/psychiatric models such as BioGPT-Large, Med-PaLM 2, MentalBERT and MentalRoBERTa, and (iii) privacy-preserving open-source models fine-tuned on clinical notes (e.g., Llama-3-Medical).

Systematically comparing these models under the same stratified data split will clarify how much *domain pre-training* and *model scale* contribute to accuracy, recall for high-risk classes (e.g., Suicidal), and explainability. This broader evaluation will address the reviewer’s concern that relying on a single generic LLM may mask the potential of specialised models and will provide a more comprehensive picture of state-of-the-art capabilities in mental-health text classification.

2. Clinically validated datasets:

Developing and utilizing datasets with professional clinical annotations would significantly enhance model reliability and facilitate more direct translation to clinical applications.

3. Neurodiverse-inclusive modeling:

Creating specialized datasets and adaptive classification frameworks that account for the unique linguistic patterns and emotional expressions characteristic of neurodiverse populations represents a critical area for enhancing inclusivity and accuracy.

4. Expanded indicator set:

Future work will broaden the label space by adding clinically relevant categories such as Autism Spectrum Disorder, ADHD (Attention-Deficit/Hyperactivity Disorder), and PTSD (Post-Traumatic Stress Disorder). Incorporating these additional indicators will enable finer-grained classification and more personalised feedback for diverse user groups.

5. Ensemble methodologies:

Integrating the complementary strengths of traditional NLP approaches and fine-tuned LLMs through ensemble frameworks offers potential for improved classification accuracy beyond what either approach achieves independently.

6. Clinical implementation studies:

Validating model predictions against standardized clinical assessments in controlled healthcare settings would establish clinically relevant performance thresholds and determine the practical utility of these systems as screening or decision-support tools.

7. Multimodal integration:

Expanding beyond text analysis to incorporate other data modalities, such as speech patterns, social media activity metrics, or digital phenotyping from smartphones, could provide a more comprehensive assessment of mental health status.

8. Longitudinal monitoring systems:

Developing frameworks capable of tracking mental health indicators over time would enable monitoring of treatment efficacy, disease progression, and early detection of condition changes or relapses.

9. Human-AI collaborative systems:

Designing interactive systems that effectively combine algorithmic classification with human clinical expertise represents a promising approach for maximizing both computational efficiency and contextual understanding while maintaining appropriate clinical oversight.

These future directions emphasize the need for interdisciplinary collaboration between computer scientists, mental health professionals, and patient advocates to develop systems that not only achieve technical excellence but also address real clinical needs while respecting ethical considerations of privacy, fairness, and inclusivity.

## Conclusion

This study demonstrates that our traditional, although optimized, NLP model outperformed the fine-tuned and prompt-engineered LLMs, achieving an impressive accuracy of 95% in classifying mental health statuses. Fine-tuning the GPT-4o-mini model to the mental health dataset significantly improved its performance compared to the prompt-engineered version, but it still fell short of the NLP model’s superior accuracy.

While the fine-tuned model offers advantages in handling more complex and nuanced mental health conditions like Suicidal ideation and Personality Disorder, the NLP model provides practical benefits, including superior accuracy and precision, data privacy, efficiency, and interpretability across multiple indicators. These strengths make the NLP model a viable alternative for applications where computational resources are limited, or data privacy is paramount.

The integration of fine-tuned LLMs with NLP methods, alongside human expertise, represents a powerful strategy for enhancing mental health monitoring and detection in digital spaces. Future work should focus on adapting these models to serve neurodiverse populations better, expanding datasets to reflect better neurodiverse populations and exploring additional model architectures that can further enhance classification accuracy, particularly for underrepresented mental health conditions. By building and fine-tuning models that reflect the diversity of mental health expressions, we can ensure more equitable and effective support for all individuals.

## Data Availability

The data supporting the findings of this study are not openly available. Anonymized data are, however, available from the corresponding author upon request.The underlying code for this study is available from the corresponding author upon request.

## References

[1] Whiteford, H. A. et al. Global burden of disease attributable to mental and substance use disorders: findings from the global burden of disease study 2010. *Lancet*. **382** (9904), 1575–1586 (2013). 23993280 10.1016/S0140-6736(13)61611-6

[2] Vigo, D., Thornicroft, G. & Atun, R. Estimating the true global burden of mental illness. *Lancet Psychiatry*. **3** (2), 171–178 (2016). 26851330 10.1016/S2215-0366(15)00505-2

[3] Kim, J., Lee, J., Park, E. & Han, J. A deep learning model for detecting mental illness from user content on social media. *Sci. Rep.* **10** (1), 11846 (2020). 32678250 10.1038/s41598-020-68764-y PMC7367301

[4] Guntuku, S. C., Yaden, D. B., Kern, M. L., Ungar, L. H. & Eichstaedt, J. C. Detecting depression and mental illness on social media: an integrative review. *Curr. Opin. Behav. Sci.* **18**, 43–49 (2017).

[5] Abd-alrazaq, A. et al. The performance of artificial intelligence-driven technologies in diagnosing mental disorders: an umbrella review. *NPJ Digit. Med.* **5**(1), 87. 10.1038/s41746-022-00631-8 (2022). 10.1038/s41746-022-00631-8 PMC9262920 35798934

[6] Chin, H. et al. The potential of chatbots for emotional support and promoting mental well-being in different cultures: mixed methods study. *J Med. Internet Res.* **25**, e51712. 10.2196/51712 (2023). 10.2196/51712 PMC10625083 37862063

[7] Gkotsis, G. et al. Characterisation of mental health conditions in social media using informed deep learning. *Sci. Rep.* **7** (1), 1–11 (2017). 28327593 10.1038/srep45141 PMC5361083

[8] Aronson, A. R. & Lang, F-M. An overview of metamap: historical perspective and recent advances. *J. Am. Med. Inf. Assoc.* **17** (3), 229–236 (2010). 10.1136/jamia.2009.002733 PMC2995713 20442139

[9] Savova, G. K. et al. Mayo clinical text analysis and knowledge extraction system (cTAKES): architecture, component evaluation and applications. *J. Am. Med. Inf. Assoc.* **17** (5), 507–513 (2010). 10.1136/jamia.2009.001560 PMC2995668 20819853

[10] Dalal, S. et al. A cross attention approach to diagnostic explainability using clinical practice guidelines for depression. *IEEE J. Biomed. Health Inform.* (2024). 10.1109/JBHI.2024.3483577 39418143

[11] Dalal, S., Jain, S. & Dave, M. Deep knowledge-infusion for explainable depression detection. *arXiv preprint arXiv:240902122* (2024).

[12] Lee, J. et al. BioBERT: a pre-trained biomedical language representation model for biomedical text mining. *Bioinformatics*. **36** (4), 1234–1240 (2020). 31501885 10.1093/bioinformatics/btz682 PMC7703786

[13] Alsentzer, E. et al. Publicly available clinical BERT embeddings. *arXiv preprint arXiv:190403323* (2019).

[14] Howard, J. & Ruder, S. Universal language model fine-tuning for text classification. *arXiv preprint arXiv:180106146* (2018).

[15] PowersDM Evaluation: from precision, recall and F-measure to ROC, informedness, markedness and correlation. *ArXiv Preprint*. arXiv:201016061 (2020).

[16] Stade, E. C., Ungar, L., Eichstaedt, J. C., Sherman, G. & Ruscio, A. M. Depression and anxiety have distinct and overlapping language patterns: results from a clinical interview. *J. Psychopathol. Clin. Sci*. **132** (8), 972–983. 10.1037/abn0000850 (2023). 37471025 10.1037/abn0000850 PMC10799169

[17] Fineberg, S. K. et al. Self-reference in psychosis and depression: a language marker of illness. *Psychol. Med.* **46** (12), 2605–2615. 10.1017/S0033291716001215 (2016). 27353541 10.1017/S0033291716001215 PMC7944937

[18] Zhang, C., Bengio, S., Hardt, M., Recht, B. & Vinyals, O. Understanding deep learning (still) requires rethinking generalization. *Commun. ACM*. **64** (3), 107–115 (2021).

[19] Yemisi, T., Richard, D. R. & Jonathan, J. D. Meta-analysis of diagnostic accuracy studies in mental health. *Evid. Based Ment. Health*. **18** (4), null. 10.1136/eb-2015-102228 (2015). 10.1136/eb-2015-102228 PMC4680179 26446042

[20] Tauscher, J. S. et al. Automated detection of cognitive distortions in text exchanges between clinicians and people with serious mental illness. *Psychiatr. Serv.* **74** (4), 407–410 (2023). 36164769 10.1176/appi.ps.202100692

